# Melatonin alleviates chronic intermittent hypoxia-induced gastric mucosal injury via attenuation of oxidative stress and JNK-mediated apoptotic signaling in rats

**DOI:** 10.1371/journal.pone.0338391

**Published:** 2026-01-13

**Authors:** Hong L. Ji, Hua L. Yu, Jia F. Luo, Xin R. Li, Cheng X. Nie, Tie J. Liu, Huan H. Jiang, Cong H. Liu, Jia B. Zhang, Xin H. Yuan, Xiao F. Song, Yue D. Li, Yanlei Ge, Ai S. Fu

**Affiliations:** 1 North China University of Science and Technology School of Clinical Medicine, Tangshan, Hebei, China; 2 Department of Respiratory Medicine, North China University of Science and Technology Affiliated Hospital, Tangshan, Hebei, China; 3 Department of Anesthesiology, North China University of Science and Technology Affiliated Hospital, Tangshan, Hebei, China; 4 Department of Pharmacy, North China University of Science and Technology Affiliated Hospital, Tangshan, Hebei, China; 5 Department of Endocrinology, North China University of Science and Technology Affiliated Hospital, Tangshan, Hebei, China; 6 Department of Otolaryngology, Hebei Provincial People’s Hospital, Shijiazhuang, Hebei, China; Helwan University, EGYPT

## Abstract

**Background:**

To investigate the mechanism of chronic intermittent hypoxia on gastric injury in rats and the intervening effect and possible mechanism of melatonin.

**Methods:**

Forty-eight male Wistar rats were randomly divided into normal control, intermittent hypoxia, and melatonin treatment groups. Subgroups (n = 4 per time point) were treated for 2, 4, 6, and 8 weeks. Gastric tissue morphology, gastric juice pH, pepsin levels, oxidative stress markers (MDA and SOD), and the expression of JNK and apoptosis-related genes (Bax, Bcl-2) were assessed.

**Results:**

The intermittent hypoxia group exhibited significant gastric mucosal damage, decreased pH, increased pepsin, elevated MDA, reduced SOD, and upregulation of JNK and Bax/Bcl-2 mRNA ratio. Melatonin treatment markedly alleviated these pathological and molecular changes compared to the intermittent hypoxia group (*P* < 0.05).

**Conclusion:**

Chronic intermittent hypoxia induces gastric mucosal injury, which is associated with oxidative stress imbalance and activation of JNK-mediated apoptotic signaling. Melatonin exerts a protective effect by enhancing antioxidant capacity and suppressing the JNK-Bax/Bcl-2 pathway.

## Introduction

Obstructive sleep apnea-hypopnea syndrome (OSAHS) characterized by recurrent nocturnal hypoxia-reoxygenation, similar to ischemia-reperfusion, is a systemic disease that can involve multiple organs throughout the body, and it is associated with a variety of respiratory disorders such as chronic cough, chronic obstructive pulmonary disease (COPD), asthma and pulmonary hypertension, etc. [1–4].OSAHS mainly causes the pathophysiological changes of sleep fragmentation and intermittent hypoxia (IH), and IH is considered to be an important pathophysiological mechanism of OSAHS as a source disease affecting the whole body. OSAHS is a chronic respiratory disease that can lead to systemic multi-system damage through oxidative stress and inflammation, but the gastric mucosal damage associated with OSAHS has not yet been reported. It has been shown that the gastric mucosa can be damaged to different degrees (acute gastric mucosal injury, acute upper gastrointestinal hemorrhage) under the low oxygen conditions of plateau through oxygen radical damage and inflammatory reaction [5]. Fang Huashan et al. simulated IH at high altitude and found that prolonged exposure to IH prolonged gastric emptying time in rats [6]. In summary, the gastric mucosa is a fragile organ, and hypoxia causes different degrees of damage to the gastric mucosa through oxidative stress, activation of signaling pathways, etc. Therefore, it is hypothesized that OSAHS may induce gastric mucosal damage.

Melatonin (MT) is the main hormone secreted by the pineal gland, which regulates circadian rhythms (sleep-wake rhythms, neuroendocrine rhythms, and temperature cycles) by acting on MT1 and MT2 receptors, protects the brain from oxidative stress by acting on MT3 receptors, participates in the autonomic regulation of blood pressure and the heart, and the regulation of the immune system [7,8]. Sleep deprivation is a difficult problem that afflicts many people today and can lead to metabolic disorders in the organism, affecting people’s quality of life. Sleep deprivation can lead to a strong stress response in the organism, resulting in systemic multi-system damage. Studies have shown that sleep deprivation can lead to gastric mucous membrane damage and a series of digestive system symptoms, while the oxidative stress indicators of the organism decreased significantly after MT treatment was given, and the above symptoms and signs were significantly reduced [9]. Wang Zhong [10] et al. showed that MT has high antioxidant properties, scavenges Reactive Oxygen Species (ROS), protects mitochondrial oxidoreductase, Superoxide Dismutase (SOD), and other important proteins and enzymes that can attenuate oxidative damage to DNA, and reduces inflammation,. thereby improving ICH-induced asymptomatic cerebral infarction. Sun Hang [11] et al. found that MT could improve high-fat diet-induced NAFLD by reducing oxidative stress and ameliorating liver injury through the MAPK-JNK signaling pathway.

Critically, the JNK pathway, a member of the MAPK family, serves as a key downstream effector of oxidative stress and can propagate cellular damage by promoting apoptosis, a process regulated by the balance between pro-apoptotic (e.g., Bax) and anti-apoptotic (e.g., Bcl-2) proteins. Whether this pathway is implicated in IH-induced gastric injury and the protective actions of MT remains unclear.

Therefore, in this study, we established an IH model to investigate the effects of IH on the gastric mucosa and to clarify the underlying mechanisms of injury. Furthermore, we verified the interventive effects of MT and explored its potential mechanisms, specifically examining its impact on oxidative stress and the JNK-mediated apoptotic pathway. This work aims to provide a theoretical foundation for the clinical prevention and treatment of OSAHS-associated gastric mucosal lesions.

## 1. Materials and methods

### 1.1 Ethics statement

This study was approved by the ethics committee of North China University of Science and Technology Affiliated Hospital (Approval number: LX2019069). All experiments were carried out in compliance with the ARRIVE guidelines. All methods were carried out in accordance with relevant guidelines and regulations.

### 1.2 Materials, reagents and instruments

48 clean-grade healthy adult male Wistar rats, weighing 190 ± 10g, purchased from Beijing Viton Lihua Laboratory Animal Science and Technology Company Limited (License No.: SCXK (Beijing) 2016−0002), hypoxia chamber (Beijing Xitianlong Co., Ltd.), program control and monitoring system (Tangshan Youyi Science and Technology Co., Ltd.), oil-free lubricated air compressor (Shanghai People’s Group Industry Co., Ltd.), oxygen meter SMART (Xiamen Xinrui Ltd), Oxygen Meter SMART (Xiamen Xinrui Instrumentation Co.),MT was purchased from Sigma-aldrich, slicer (Leica, Germany), blood gas analyzer (Central Laboratory of North China University of Science and Technology), superoxide dismutase (SOD) kit, malondialdehyde (MDA) kit, and BCA protein concentration determination kit (AndyGene Biotechnology Co., Ltd.), Reverse transcription instrument (BIO RAD, USA), PCR instrument (Eppendorf, Germany), Rotor-Gene3000 analysis software (Eppendorf, Germany), real-time fluorescence quantitative polymerase chain reaction instrument (Leica, Germany), RNA primer (Beijing Ruibo Xingke Biotechnology Co., Ltd.), RNA reverse transcription kit (Beijing Zhuangmeng International Biogenetic Technology Co., Ltd.), total RNA extraction kit (Wuhan U-Test Biotechnology Co., Ltd.), PCR amplification kit (Beijing Zhuangmeng International Biogenetic Technology Co., Ltd.). RNA extraction kit (Wuhan U-Test Biotechnology Co., Ltd.), PCR amplification kit (Beijing Zhuangmeng International Biogenetic Technology Co., Ltd.).

Configuration of experimental reagents

1) MT injection: 100 mg of MT was dissolved in 2 ml of anhydrous ethanol, and after it was fully dissolved, then 62 ml of 0.9% sodium chloride was added into it to make it fully mixed.
2) 4% paraformaldehyde: 40g paraformaldehyde powder + 0.02 mol/L 1000 ml PBS, at the same time, add about 10g sodium hydroxide to the solution, adjust the PH to about 7.4, and make it fully dissolved with magnetic stirrer.
3) 20% Ulatan: 20g Ulatan powder + 100 ml 0.9% sodium chloride, make it mix well.

### 1.3 Animal grouping and model preparation

Forty-eight male Wistar rats were randomly divided into three groups using a randomized number table: the normal control group (NC group, n = 16), the IH group (IH group, n = 16), and the melatonin treatment group (MT group, n = 16). The experiment lasted for 8 weeks, with subgroups (n = 4) assessed at 2, 4, 6, and 8 weeks.

Drug Administration: Daily intraperitoneal injections were administered at 7:30. The MT group received melatonin (10 mg/kg) [12,13]. The NC and IH groups received an equal volume of the ethanol-saline vehicle. Doses were adjusted weekly based on body weight.

IH Protocol: From 8:00–15:00 daily, rats in the IH and MT groups were placed in an IH chamber. The chamber underwent a 120-second cycle, alternating between nitrogen and compressed air, to maintain oxygen concentration between 5% and 21%. Rats in the NC group were housed in an identical chamber with a continuous flow of compressed air for the same period.

All rats were fed normally after the daily experimental procedures.

### 1.4 Tissue treatment

At the end of each time point (2, 4, 6, or 8 weeks), rats were humanely euthanized. Following the daily experimental procedures, rats were deeply anesthetized with an intraperitoneal injection of 20% urethane (0.5 ml/100g). The depth of anesthesia was confirmed by the absence of a pedal withdrawal reflex and no reaction to a tail pinch. No analgesics were used as this was a terminal procedure. Once surgical anesthesia was confirmed, rats were fixed in a supine position. The cardia and pylorus of the stomach were ligated, and the entire stomach was removed. Death was confirmed by cervical dislocation following the laparotomy and tissue collection.

#### Gastric juice analysis.

Gastric contents were collected into EP tubes, centrifuged at 3000 r/min for 10 min, and the supernatant was used for pH and pepsin level measurement.

#### Gastric tissue analysis.

The stomach was cut along the greater curvature, rinsed with saline, and photographed. To ensure consistency, all tissue samples for subsequent analysis (histology, molecular biology) were systematically obtained from the same anatomical region—the mid-glandular portion of the gastric corpus, along the central wall of the stomach body. These mucosal samples were then divided; one part was fixed in 4% paraformaldehyde for HE staining and immunohistochemistry, and the other was snap-frozen for RNA analysis.

### 1.5 Determination of gastric fluid pH and pepsin

#### pH measurement.

The pH of gastric juice was directly measured using a desktop PHS-3C precision pH meter.

#### Pepsin measurement.

Pepsin levels were determined using a double-antibody sandwich ELISA kit according to the manufacturer’s instructions. Absorbance was measured at 450 nm, and pepsin concentration was calculated based on the standard curve.

### 1.6 Hematoxylin-eosin staining of IH rat gastric tissue

Fixed gastric tissues from the corpus region were processed routinely: paraffin-embedded, sectioned to 6 μm thickness, baked, deparaffinized, and rehydrated. Sections were stained with hematoxylin for 6 min, differentiated in 2% hydrochloric acid alcohol, rinsed, counterstained with eosin for 3 min, dehydrated, cleared, and mounted. Morphological changes were observed and photographed under a light microscope. A semi-quantitative assessment of histopathological damage (inflammatory cell infiltration, gland structure integrity, hemorrhage) was performed by a pathologist blinded to the groups.

### 1.7 Determination of SOD and MDA content

Gastric tissue samples were weighed, homogenized in cold PBS (pH 7.4), and centrifuged at 3000 r/min for 20 min. The supernatant was collected. MDA concentration and SOD activity were measured using commercial kits according to the manufacturer’s instructions, and the optical density was read with an enzyme-labeled instrument.

### 1.8 Analysis of apoptosis-related mRNA expression by quantitative Real-Time PCR (qRT-PCR)

Gastric mucosal RNA was extracted with TRIzol. cDNA was synthesized using a reverse transcription kit. qRT-PCR was performed using SYBR Green Master Mix on an ABI QuantStudio system. The primer sequences are listed in Table 1. The relative mRNA expression levels were calculated using the 2^–ΔΔCt method, with GAPDH as the endogenous control. The Bax/Bcl-2 mRNA ratio was calculated.

**Table 1:** PH value of gastric juice in three groups of rats at different time points.

Gene	Primer orientation：5' to 3'	Primer length
GAPDH	Upstream: ACTCTACCCACGGCAAGTTC	72bp
	Downstream: TGGGTTTCCCGTTGATGACC	
JNK	Upstream: CCAAGAGCCTGAACTTTCCCA	89bp
	Downstream: GTGCTCAACAGACTGACTGC	
Bax	Upstream: CCAAGAGCCTGAACTTTCCCA	314bp
	Downstream: GGGTCCCGAAGTAGGAAAGG	
Bcl-2	Upstream: AGCATGCGACCTCTGTTTGA	108bp
	Downstream: TCACTTGTGGCCCAGGTATG	

Note: The groups in the table have n = 4 test animals at each observation time point, *: *P* < 0.05, compared with NC group; ^#^: *P* < 0.05, compared with IH group.

### 1.9 Statistical processing

Data were statistically analyzed using SPSS 22.0 statistical software, and the measurement data conformed to normal distribution and variance chi, and the data were expressed as “mean±standard deviation” (x ± s), and the data in each group and at different time points in each index were analyzed using two-factor analysis of variance (ANOVA), and the data in each index were compared using two-by-two comparison with LSD post hoc, and the difference was considered statistically significant when *P* < 0.05. 0.05 was taken as the difference being statistically significant.

## 2 Results

### 2.1 Histopathological changes of gastric mucosa in three groups of rats

**Fig 1:**
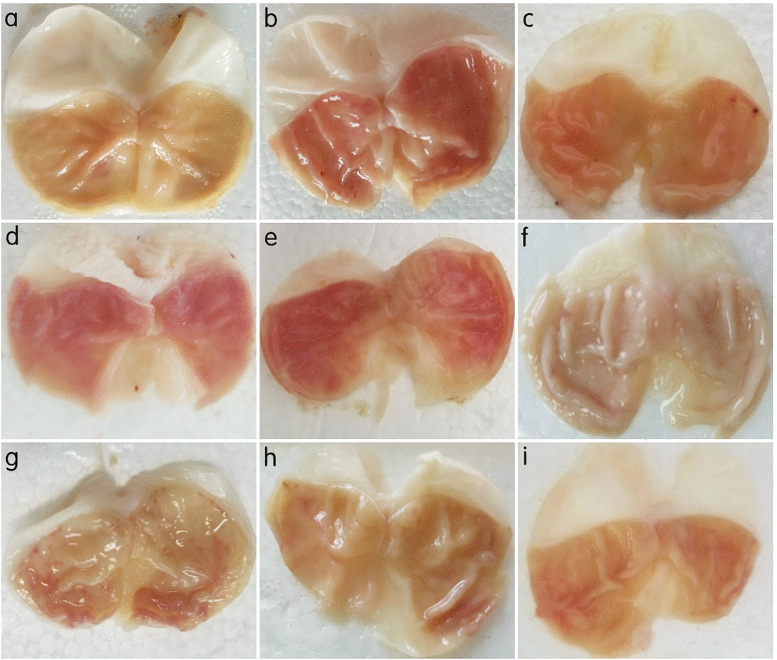
Changes of gastric mucosa in three groups of rats. Note: a: normal control; b: Intermittent Hypoxia(IH) 2w; c: IH 4w; d: IH 6w; e: IH 8w; f: Melatonin(MT) 2w; g: MT 4w; h: MT 6w; i: MT 8w.

Macroscopic observations: The gastric mucosa surface in the NC group was smooth with neat and clear folds at all time points. In contrast, the IH and MT groups showed varying degrees of hemorrhage, erosion, and ulceration. The damage worsened with prolonged IH exposure. However, the MT group exhibited less severe damage compared to the IH group at each corresponding time point, as shown in Fig 1a-1i.

**Fig 2:**
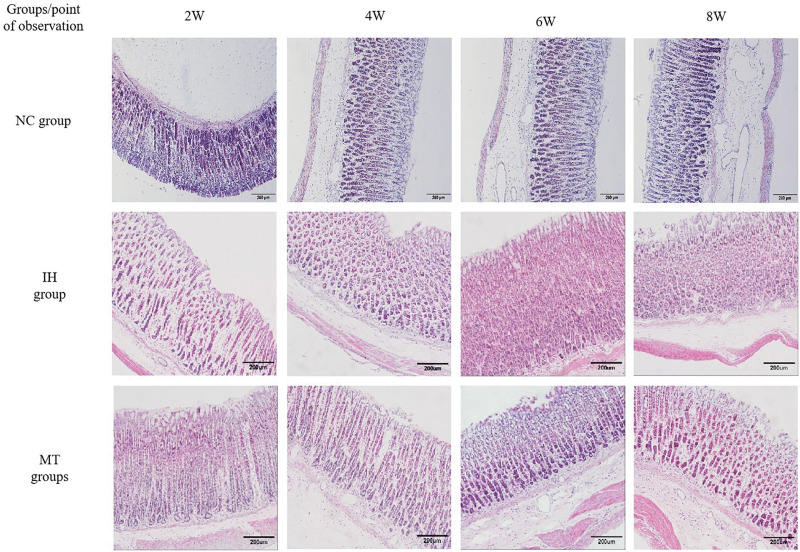
Cytopathologic changes in the gastric mucosal tissue of rats in three groups (HE staining, × 100).

Microscopic observations (HE staining): Gastric mucosal glands in the NC group were structurally intact with regularly arranged cells. The IH group showed inflammatory cell infiltration (neutrophils, lymphocytes, plasma cells), hemorrhage around gastric glands, and disrupted glandular structure, which worsened over time. These pathological changes were markedly reduced in the MT group compared to the IH group as shown in Fig 2.

### 2.2 Comparison of gastric juice pH among three groups of rats

**Fig 3:**
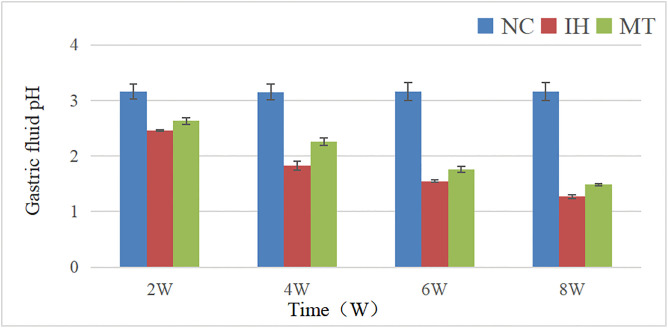
The pH value of gastric juice was compared in three groups.

As shown in Table 2 and Fig 3, two-factor ANOVA showed that the differences in rat gastric juice pH between groups and time were statistically significant (F between groups (2, 36) = 1167.078, *P* = 0.000; F time (3, 36) = 181.653, *P* = 0.000), and it can be assumed that the gastric juice pH values of the different groups were unequal or incomplete in relation to the respective observation time points. Comparison between the different groups showed that the gastric fluid PH values in the IH and MT groups were lower than those in the NC group (*P* < 0.05);Comparison of gastric fluid pH at each observation time point showed a gradual decrease with increasing time (*P* < 0.05). The interaction between groups and time (F between groups * time (6, 36) = 48.241, *P* = 0.000) had a significant effect on gastric juice pH.Post hoc analysis showed that the gastric juice pH at each observation time point in the IH and MT groups were lower than that in the NC group, respectively (*P* < 0.05).The gastric juice pH at each observation time point in the MT group was higher than that in the IH group, respectively (*P* < 0.05).

**Table 2:** PH value of gastric juice in three groups of rats at different time points.

groups	point of observation			
	2w	4w	6w	8w
NC group	3.17 ± 0.11	3.15 ± 0.12	3.16 ± 0.13	3.18 ± 0.14
IH group	2.46 ± 0.04^*^	1.86 ± 0.09^*^	1.54 ± 0.03^*^	1.28 ± 0.04^*^
MT group	2.61 ± 0.07^*#^	2.29 ± 0.08^*#^	1.77 ± 0.05^*#^	1.48 ± 0.03^*#^

Note: The groups in the table have n = 4 test animals at each observation time point, *: *P* < 0.05, compared with NC group; ^#^: *P* < 0.05, compared with IH group.

### 2.3 Comparison of pepsin levels in three groups of rats

**Fig 4:**
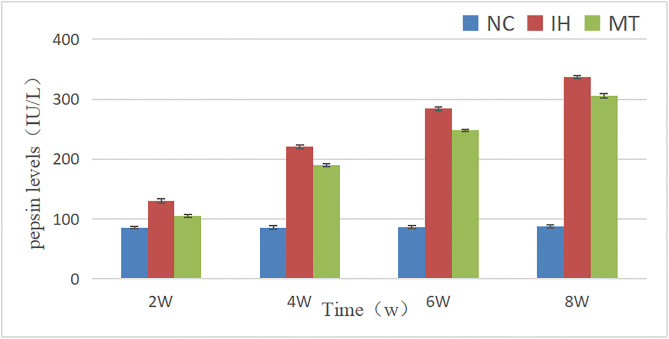
Comparison of pepsin levels in three groups at different time.

As shown in Table 3 and Fig 4, two-factor ANOVA showed that the differences in rat gastric fluid pepsin levels between groups and time were statistically significant (F between groups (2, 36) = 14,283.073, *P* = 0.000; F time (3, 36) = 5,325.223, *P* = 0.000), and it can be assumed that the pepsin levels of the different groups were unequal or incomplete with respect to each observation time point. Comparison between different groups showed that pepsin levels in the IH and MT groups were higher than those in the NC group (*P* < 0.05); comparison between the various observation time points showed that pepsin levels gradually increased with increasing time (*P* < 0.05).The interaction between groups and time (F between groups * time (6, 36) = 1291.922, *P* = 0.000) had a significant effect on pepsin levels.Post hoc analysis showed that the pepsin levels at each observation time point in the IH and MT groups were higher than those in the NC group, respectively, and the differences were statistically significant (*P* < 0.05).The pepsin levels at each observation time point in the MT group were lower than those in the IH group, and the differences were statistically significant (*P* < 0.05).

**Table 3:** The levels of pepsin in three groups of rats at different time (IU/L).

groups	point of observation			
	2w	4w	6w	8w
NC group	85.38 ± 1.98	85.65 ± 2.83	86.62 ± 2.54	87.54 ± 3.04
IH group	130.02 ± 3.57^*^	220.79 ± 3.15^*^	284.19 ± 2.71^*^	336.9 ± 2.56^*^
MT group	105.02 ± 2.38^*#^	189.56 ± 2.36^*#^	247.92 ± 2.09^*#^	305.42 ± 3.58^*#^

Note: The groups in the table have n = 4 test animals at each observation time point, *: *P* < 0.05, compared with NC group; ^#^: *P* < 0.05, compared with IH group.

### 2.4 MDA content and SOD activity in gastric tissues of rats in three groups

**Fig 5:**
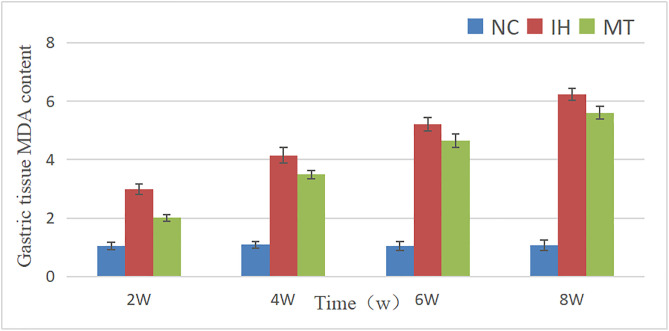
Comparison of MDA levels in gastric tissues of three groups at different time.

**Fig 6:**
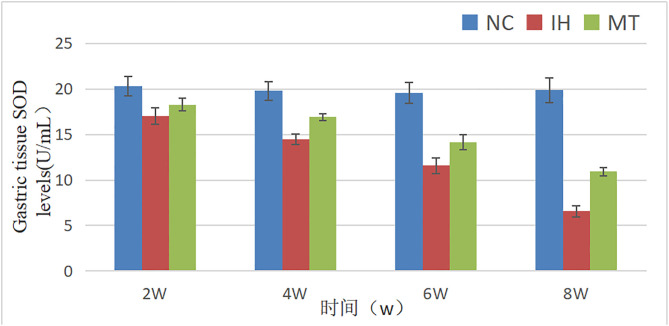
Comparison of SOD levels in gastric tissues of three groups at different time.

Comparison between different groups showed that the MDA content at each observation point in IH and MT groups was higher than that in NC group respectively, and with the increase of hypoxia time, the MDA level in gastric tissues gradually increased, and the MDA content at each observation point in MT group was significantly lower than that in IH group respectively (*P* < 0.05);Compared with the NC group, the SOD levels at each observation point in the IH and MT groups were significantly lower (*P* < 0.05), and the SOD levels at each observation point in the MT group were higher than those in the IH group, respectively, suggesting that MT can reduce the level of MDA in gastric tissues and increase SOD activity (*P* < 0.05). See Tables 4 and 5, Figs 5 and 6.

**Table 4:** Serum MDA content in three groups at different time (nmol/L).

groups	point of observation			
	2w	4w	6w	8w
NC group	1.05 ± 0.12	1.09 ± 0.12	1.05 ± 0.16	1.08 ± 0.18
IH group	2.99 ± 0.19^*^	4.15 ± 0.26^*^	5.2 ± 0.23^*^	6.23 ± 0.21^*^
MT group	2.01 ± 0.11^*#^	3.49 ± 0.14^*#^	4.65 ± 0.24^*#^	5.6 ± 0.22^*#^

Note: The groups in the table have n = 4 test animals at each observation time point, *: *P* < 0.05, compared with NC group; ^#^: *P* < 0.05, compared with IH group.

**Table 5:** Serum SOD activity in three groups of rats at different time (U/mL).

groups	point of observation			
	2w	4w	6w	8w
NC group	20.27 ± 1.07	19.77 ± 1.04	19.55 ± 1.14	19.87 ± 1.35
IH group	17.04 ± 0.92^*^	14.48 ± 0.61^*^	11.58 ± 0.86^*^	6.59 ± 0.61^*^
MT group	18.27 ± 0.7^*#^	16.9 ± 0.38^*#^	14.14 ± 0.84^*#^	10.91 ± 0.46^*#^

Note: The groups in the table have n = 4 test animals at each observation time point, *: *P* < 0.05, compared with NC group; ^#^: *P* < 0.05, compared with IH group.

### 2.5 Expression of JNK and apoptosis-related genes in gastric tissues

To elucidate the molecular mechanisms underlying IH-induced gastric injury and MT protection, we assessed the mRNA expression of JNK, a key stress-responsive kinase, and the apoptosis-related genes Bax and Bcl-2.

**Fig 7:**
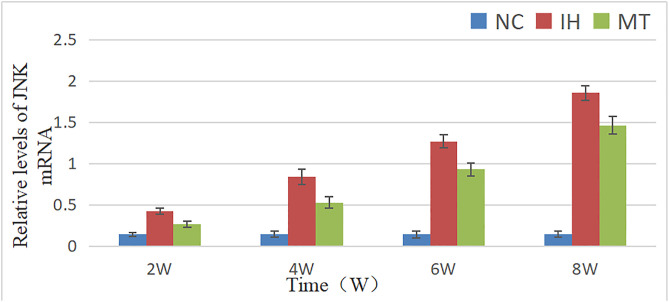
Comparison of the relative expression of JNK mRNA in gastric mucosa of three groups of rats.

The mRNA expression of JNK was significantly influenced by group, time, and their interaction (F between groups (2, 36) = 890.326, *P* = 0.000; F time (3, 36) = 402.17, *P* = 0.000; F interaction (6, 36) = 102.363, *P* = 0.000). Post hoc analysis revealed that JNK mRNA levels in the IH and MT groups were significantly higher than those in the NC group at all time points (*P* < 0.05). Notably, MT treatment significantly attenuated this IH-induced upregulation, as evidenced by the lower JNK mRNA levels in the MT group compared to the IH group at each corresponding time point (*P* < 0.05), As shown in Table 6 and Fig 7.

**Table 6:** Relative levels of JNK mRNA in gastric mucosa of the three groups.

groups	point of observation			
	2w	4w	6w	8w
NC group	0.143 ± 0.025	0.148 ± 0.035	0.143 ± 0.039	0.146 ± 0.04
IH group	0.423 ± 0.038^*^	0.838 ± 0.09^*^	1.27 ± 0.08^*^	1.855 ± 0.087^*^
MT group	0.27 ± 0.037^*#^	0.53 ± 0.066^*#^	0.928 ± 0.075^*#^	1.463 ± 0.106^*#^

Note: The groups in the table have n = 4 test animals at each observation time point, *: *P* < 0.05, compared with NC group; ^#^: *P* < 0.05, compared with IH group.

**Fig 8:**
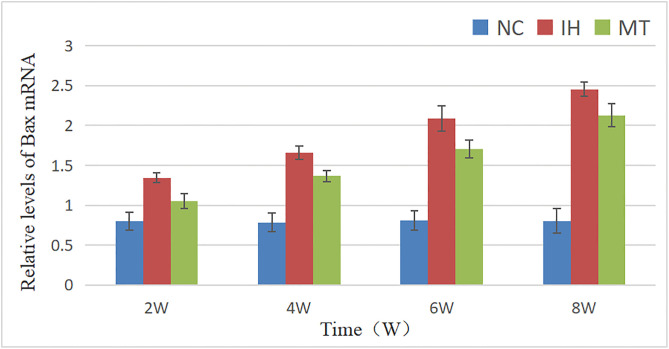
Comparison of Bax mRNA expression in gastric mucosa of three groups of rats.

**Fig 9:**
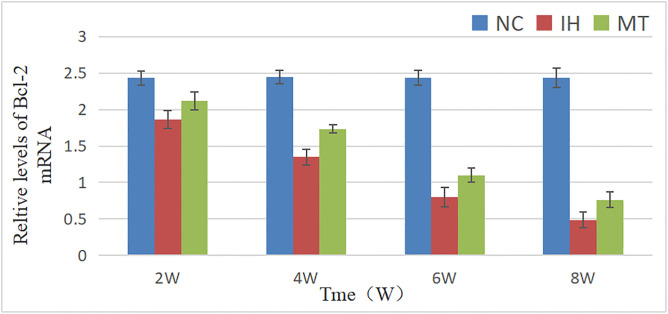
Comparison of relative expression of bcl-2 mRNA in gastric mucosa of three groups of rats.

Similarly, the mRNA expression of Bax and Bcl-2 were significantly affected by group, time, and their interaction (for Bax: F between groups (2, 36) = 387.878, *P* = 0.000; F time (3, 36) = 94.148, *P* = 0.000; F interaction (6, 36) = 23.03, *P* = 0.000. For Bcl-2: F between groups (2, 36) = 657.505, *P* = 0.000; F time (3, 36) = 172.37, *P* = 0.000; F interaction (6, 36) = 43.214, *P* = 0.000). The IH group exhibited a pro-apoptotic gene expression profile, characterized by a significant increase in Bax mRNA and a decrease in Bcl-2 mRNA compared to the NC group (*P* < 0.05). MT intervention effectively counteracted these changes, resulting in lower Bax mRNA and higher Bcl-2 mRNA levels compared to the IH group (*P* < 0.05), As shown in See Table 7, Fig 8 and Table 8, Fig 9.

**Table 7:** Relative levels of Bax mRNA in gastric mucosa of the three groups.

groups	point of observation			
	2w	4w	6w	8w
NC group	0.803 ± 0.111	0.785 ± 0.119	0.81 ± 0.124	0.805 ± 0.153
IH group	1.345 ± 0.06^*^	1.658 ± 0.081^*^	2.09 ± 0.16^*^	2.453 ± 0.088^*^
MT group	1.053 ± 0.091^*#^	1.368 ± 0.07^*#^	1.705 ± 0.108^*#^	2.13 ± 0.142^*#^

Note: The groups in the table have n = 4 test animals at each observation time point, *: *P* < 0.05, compared with NC group; ^#^: *P* < 0.05, compared with IH group.

**Table 8:** Relative levels of Bcl-2 mRNA in gastric mucosa of the three groups.

groups	point of observation			
	2w	4w	6w	8w
NC group	2.433 ± 0.095	2.445 ± 0.096	2.432 ± 0.103	2.435 ± 0.128
IH group	1.865 ± 0.122^*^	1.348 ± 0.107^*^	0.803 ± 0.132^*^	0.488 ± 0.103^*^
MT group	2.115 ± 0.122^*#^	1.735 ± 0.056^*#^	1.1 ± 0.096^*#^	0.763 ± 0.104^*#^

Note: The groups in the table have n = 4 test animals at each observation time point, *: *P* < 0.05, compared with NC group; ^#^: *P* < 0.05, compared with IH group.

## Discussion

OSAHS is a common and frequent clinical disease, which can cause systemic multi-system diseases, and 40% to 50% of OSAHS patients have complications, which cause serious harm to the human body, and clinical statistics show that the 5-year morbidity and mortality rate of untreated OSAHS patients reaches 11% to 13%, and the gastric mucosa will inevitably be involved [14]. In the present study, using the IH model, we found that IH directly caused gastric mucosal injury in rats, which worsened over time, suggesting that IH is a key mechanism leading to OSAHS-associated gastropathy. In this study, we observed the protective effect of MT on gastric mucosal injury in rats by simulating the nocturnal IH in patients with OSAHS. it was found that IH induced gastric mucosal injury in rats, which was characterised by not only histopathological lesions and secretory dysfunction, but also accompanied by a well-defined molecular signature of oxidative stress and pro-apoptotic signalling. mT treatment could protect this gastric mucosa from damage by attenuating oxidative stress and inhibiting the JNK-mediated apoptotic signalling pathway, resulting in a significant protective effect against this injury.

Studies have shown that the PH value of gastric juice can be used as a function to understand the function of gastric acid secretion and further make a judgement on the condition, as well as a guide to the next step of treatment [15–17]. Pepsinogen is produced and stored by gastric host cells and is the precursor of active pepsin, which is highly expressed in peptic ulcers [18]. It has been found that serum pepsinogen can be used as a “serological biopsy” of gastric mucosal lesions, which can sensitively reflect the functional and morphological changes of the gastric mucosa [19]. The results of the present study confirmed that IH exposure resulted in progressive gastric mucosal damage, accompanied by a decrease in gastric juice pH and an increase in pepsin levels. This has a potential clinical correspondence with common gastrointestinal symptoms (e.g., gastroesophageal reflux) in patients with OSAHS, suggesting that IH is an important pathophysiological mechanism for OSAHS-associated gastric mucosal lesions.

The gastrointestinal tract is a digestive organ that is highly susceptible to ROS attack, and ROS can cause tissue and organ damage by regulating a large number of redox-sensitive signalling pathways (e.g., the JNK pathway, the Fas pathway, and the NF-kB pathway), leading to apoptosis [20]. Mitsuyama [21] et al. found in a gastric injury model that the JNK signalling pathway was markedly expressed, and the administration of a specific JNK inhibitors significantly reduced gastric injury in rats, suggesting that activation of the JNK signalling pathway plays a key role in gastric injury. Therefore, we experimented to understand the mechanism of IH on gastric mucosal lesions by establishing an OSHSA model and detecting the expression of JNK signalling pathway-related factors: JNK, Bax, Bcl-2 mRNA in rat gastric mucosal tissues. In this study, we found that compared with the normal control group, the levels of JNK and Bax mRNA in the gastric tissues of rats in the IH group were significantly higher and the level of Bcl-2 mRNA was significantly lower, and the difference was statistically different as the duration of hypoxia was prolonged (*P* < 0.05). The results of this study indicated that OSA-induced gastric mucosal damage could be caused by activating the JNK signalling pathway, and the more activated the more obvious with the extension of hypoxia time, the more serious the gastric mucosal damage, which was consistent with the findings of Mitsuyama [19] et al.

MT is an endogenous hormone in the human body, which has the functions of antioxidant damage, anti-aging, sleep promotion, immunomodulation, and antitumour [22, 23]. As a very strong antioxidant, MT not only inhibits the generation and scavenging of free radicals, but also activates antioxidant enzymes and repairs oxidized molecules, thus improving lipid metabolism, scavenging free radicals, and increasing the level of SOD [23,24]. In the present study, by giving MT intervention to rats, the MT group showed a slight reduction of cellular damage in the gastric mucosa in the naked eye and light microscopy, an increase in the pH of gastric juice, and a decrease in the level of pepsin as compared to the IH group. In the stress ulcer model, SOD levels were found to be reduced in rats, and after administration of intraperitoneal injection of MT, SOD levels were found to be significantly increased in rats injected with MT, further indicating that MT has free radical scavenging and antioxidant functions [25]. In the present study, by giving rats MT intervention, the levels of ROS and MDA, indicators of oxidative stress in gastric mucosal tissues in the MT group were reduced and the level of SOD was increased compared with that in the IH group, with a statistically significant difference (*P* < 0.05). This indicates that MT can scavenge oxygen free radicals and enhance the body’s ability to resist oxidative stress. Meanwhile, it was also found that after MT intervention in rats, compared with the IH group, the expression of JNK mRNA, pro-apoptotic Bax mRNA, and anti-apoptotic Bcl-2 mRNA in the gastric mucosa tissue of the MT group was down-regulated, and there was a statistically significant difference between the two groups (*P* < 0.05). It indicated that after MT intervention, the organism was able to resist IH-induced oxidative stress, apoptosis, and improved the antioxidant capacity of the organism, and therefore was able to resist IH-induced gastric mucosal damage. The above findings suggest that MT may play a protective role against IH-induced gastric mucosal injury by improving the body’s free radical scavenging and antioxidant capacity and regulating the JNK pathway.

However, we must consider several limitations of this study. Firstly, the small sample size of n = 4 per time point may affect the generalisability of the results, which needs to be confirmed by future studies with larger sample sizes. Second, the study did not consider the effect of MT dose on sleep and its toxic side effects, which may have potentially biased the results. MT is an endogenous sleep-regulating hormone, and exogenous supplementation may have a direct effect on the sleep-wake cycle to promote sleep.A dose of MT of 10 mg/kg may improve the quality of improved sleep in rats to some extent, and future studies monitored by electroencephalogram (EEG) or follow-up studies using MT receptor-deficient animals could help to analyse the sleep-promoting and direct antioxidant effects of MT. Third, the study did not introduce another common antioxidant (e.g., vitamin E) as a positive control, so it is not possible to clearly distinguish whether the protective effect of MT is specific to its antioxidant mechanism or whether other antioxidants have a similar effect, and follow-up studies should include an additional antioxidant control to clarify whether the observed protective effect is specific to the multifaceted action of melatonin or a general antioxidant response. This is an important consideration for the design of future studies. Finally, translation of these promising findings from animal models to human OSAHS patients requires careful consideration. Future clinical studies should address challenges related to effective dosing (e.g., oral administration), long-term efficacy and safety of MT in patients with OSAHS, and identification of patient subgroups most likely to benefit from this adjuvant therapy.

## Conclusion

In summary, IH can cause gastric mucosal damage through the imbalance between oxidative stress and anti-oxidative stress systems, leading to gastric mucosal secretory dysfunction. MT can reduce cellular damage by scavenging oxygen radicals and improving antioxidant capacity, which provides a new idea for the treatment of OSAHS-associated gastric mucosal injury.

## References

[1] West SD, Turnbull C. Obstructive sleep apnoea. Eye (Lond). 2018;32(5):889–903. doi: 10.1038/s41433-017-0006-y 29391572 PMC5944625

[2] Osman AM, Carter SG, Carberry JC, Eckert DJ. Obstructive sleep apnea: current perspectives. Nat Sci Sleep. 2018;10:21–34. doi: 10.2147/NSS.S124657 29416383 PMC5789079

[3] Mokhlesi B, Ham SA, Gozal D. The effect of sex and age on the comorbidity burden of OSA: an observational analysis from a large nationwide US health claims database. Eur Respir J. 2016;47(4):1162–9. doi: 10.1183/13993003.01618-2015 26797029

[4] Lu M, Fang F, Wang Z, Wei P, Hu C, Wei Y. Association between serum/plasma levels of adiponectin and obstructive sleep apnea hypopnea syndrome: a meta-analysis. Lipids Health Dis. 2019;18(1):30. doi: 10.1186/s12944-019-0973-z 30684961 PMC6347767

[5] Appleton SL, Vakulin A, McEvoy RD, Vincent A, Martin SA, Grant JF, et al. Undiagnosed obstructive sleep apnea is independently associated with reductions in quality of life in middle-aged, but not elderly men of a population cohort. Sleep Breath. 2015;19(4):1309–16. doi: 10.1007/s11325-015-1171-5 25896898

[6] Fang HS, Chen CF. Influence of long-term intermittent exposures to low oxygen tensions on gastric emptying time during hypoxia. Environ Res. 1976;11(1):135–7. doi: 10.1016/0013-9351(76)90117-1 1253769

[7] Tordjman S, Chokron S, Delorme R, Charrier A, Bellissant E, Jaafari N, et al. Melatonin: Pharmacology, functions and therapeutic benefits. Curr Neuropharmacol. 2017;15(3):434–43. doi: 10.2174/1570159X14666161228122115 28503116 PMC5405617

[8] Samanta S. Physiological and pharmacological perspectives of melatonin. Arch Physiol Biochem. 2022;128(5):1346–67. doi: 10.1080/13813455.2020.1770799 32520581

[9] Han J, Wang Z, Liu Y. Protective effects of melatonin on gastric mucosal injury in sleep-deprived rats. Journal of Zhengzhou University (Medical Sciences). 2013;48(5):769–72.

[10] Wang Z, Zhou F, Dou Y, Tian X, Liu C, Li H, et al. Melatonin alleviates intracerebral hemorrhage-induced secondary brain injury in rats via suppressing apoptosis, inflammation, oxidative stress, DNA damage, and mitochondria injury. Transl Stroke Res. 2018;9(1):74–91. doi: 10.1007/s12975-017-0559-x 28766251 PMC5750335

[11] Sun H, Wang X, Chen J, Song K, Gusdon AM, Li L, et al. Melatonin improves non-alcoholic fatty liver disease via MAPK-JNK/P38 signaling in high-fat-diet-induced obese mice. Lipids Health Dis. 2016;15(1):202. doi: 10.1186/s12944-016-0370-9 27876064 PMC5120511

[12] Yuan X, Xi Z, Yu B-. Protective effect of melatonin as an antioxidant in the intestine of rats with superior mesenteric arterial occlusion. Cell Mol Biol (Noisy-le-grand). 2022;67(4):340–5. doi: 10.14715/cmb/2021.67.4.39 35809270

[13] Liu Y, Tipoe GL, Fung ML. Melatonin attenuates intermittent hypoxia-induced lipid peroxidation and local inflammation in rat adrenal medulla. Int J Mol Sci. 2014;15(10):18437–52. doi: 10.3390/ijms151018437 25314303 PMC4227224

[14] Yan W, Zhou J, Jiang M, Kong Y, Qin H, Qi Y, et al. Obstructive sleep apnea and 19 gastrointestinal diseases: a Mendelian randomization study. Front Psychiatry. 2024;15:1256116. doi: 10.3389/fpsyt.2024.1256116 39132315 PMC11310136

[15] Chinese Society of Gastroenterology, Cancer Collaboration Group of Chinese Society of Gastroenterology, Chinese Medical Association. Guidelines for diagnosis and treatment of chronic gastritis in China (2022, Shanghai). J Dig Dis. 2023;24(3):150–80. doi: 10.1111/1751-2980.13193 37245073

[16] Huang M, Liu Q, Cai J. Detection of pH, GLP and LGC in 380 cases of chronic gastric and duodenal diseases. Shenzhen Journal of Integrated Traditional Chinese and Western Medicine. 1997;7(1):25–6.

[17] Mwafy SN, Afana WM. Hematological parameters, serum iron and vitamin B12 levels in hospitalized Palestinian adult patients infected with Helicobacter pylori: a case-control study. Hematol Transfus Cell Ther. 2018;40(2):160–5. doi: 10.1016/j.htct.2017.11.010 30057990 PMC6001929

[18] Roberts NB. Review article: human pepsins - their multiplicity, function and role in reflux disease. Aliment Pharmacol Ther. 2006;24 Suppl 2:2–9. doi: 10.1111/j.1365-2036.2006.03038.x 16939427

[19] Chen C, Zhang L, Wang H. Correlation study of serum pepsin in chronic gastric lesions. Journal of Clinical Digestive Diseases. 2018;30(6):400–3.

[20] Bhattacharyya A, Chattopadhyay R, Mitra S, Crowe SE. Oxidative stress: an essential factor in the pathogenesis of gastrointestinal mucosal diseases. Physiol Rev. 2014;94(2):329–54. doi: 10.1152/physrev.00040.2012 24692350 PMC4044300

[21] Mitsuyama K, Tsuruta O, Matsui Y, Harada K, Tomiyasu N, Suzuki A, et al. Activation of c-Jun N-terminal kinase (JNK) signalling in experimentally induced gastric lesions in rats. Clin Exp Immunol. 2006;143(1):24–9. doi: 10.1111/j.1365-2249.2005.02959.x 16367930 PMC1809559

[22] Talib WH, Alsayed AR, Abuawad A, Daoud S, Mahmod AI. Melatonin in cancer treatment: current knowledge and future opportunities. Molecules. 2021;26(9):2506. doi: 10.3390/molecules26092506 33923028 PMC8123278

[23] Mortezaee K, Potes Y, Mirtavoos-Mahyari H, Motevaseli E, Shabeeb D, Musa AE, et al. Boosting immune system against cancer by melatonin: A mechanistic viewpoint. Life Sci. 2019;238:116960. doi: 10.1016/j.lfs.2019.116960 31629760

[24] Feng T-Y, Li Q, Ren F, Xi H-M, Lv D-L, Li Y, et al. Melatonin protects goat spermatogonial stem cells against oxidative damage during cryopreservation by improving antioxidant capacity and inhibiting mitochondrial apoptosis pathway. Oxid Med Cell Longev. 2020;2020:5954635. doi: 10.1155/2020/5954635 33488926 PMC7790556

[25] Hristova M, Tasinov O, Tzaneva M, Chivchibashi D, Kiselova-Kaneva Y, Bekyarova G. Effect of melatonin on the gastric antioxidant defence in experimental burn trauma. Vet Med (Praha). 2022 May 1;67(7):379–86. doi: 10.17221/109/2021-VETMED 39100131 PMC11295877

